# Cryopreservation of Induced Pluripotent Stem Cell-Derived Dopaminergic Neurospheres for Clinical Application

**DOI:** 10.3233/JPD-212934

**Published:** 2022-04-05

**Authors:** Satoe Hiramatsu, Asuka Morizane, Tetsuhiro Kikuchi, Daisuke Doi, Kenji Yoshida, Jun Takahashi

**Affiliations:** a Department of Clinical Application, Center for iPS Cell Research and Application, Kyoto University, Kyoto, Japan; b Regenerative and Cellular Medicine Kobe Center, Sumitomo Dainippon Pharma Co., Ltd, Kobe, Japan

**Keywords:** Cryopreservation, cell-based therapy, neurosphere, dopaminergic neuron, induced pluripotent stem cells, Parkinson’s disease

## Abstract

**Background:**

Pluripotent stem cell (PSC)-derived dopaminergic (DA) neurons are an expected source of cell therapy for Parkinson’s disease. The transplantation of cell aggregates or neurospheres, instead of a single cell suspension has several advantages, such as keeping the 3D structure of the donor cells and ease of handling. For this PSC-based therapy to become a widely available treatment, cryopreservation of the final product is critical in the manufacturing process. However, cryopreserving cell aggregates is more complicated than cryopreserving single cell suspensions. Previous studies showed poor survival of the DA neurons after the transplantation of cryopreserved fetal ventral-mesencephalic tissues.

**Objective:**

To achieve the cryopreservation of induced pluripotent stem cell (iPSC)-derived DA neurospheres toward clinical application.

**Methods:**

We cryopreserved iPSC-derived DA neurospheres in various clinically applicable cryopreservation media and freezing protocols and assessed viability and neurite extension. We evaluated the population and neuronal function of cryopreserved cells by the selected method *in vitro*. We also injected the cells into 6-hydroxydopamine (6-OHDA) lesioned rats, and assessed their survival, maturation and function *in vivo.*

**Results:**

The iPSC-derived DA neurospheres cryopreserved by Proton Freezer in the cryopreservation medium Bambanker hRM (BBK) showed favorable viability after thawing and had equivalent expression of DA-specific markers, dopamine secretion, and electrophysiological activity as fresh spheres. When transplanted into 6-OHDA-lesioned rats, the cryopreserved cells survived and differentiated into mature DA neurons, resulting in improved abnormal rotational behavior.

**Conclusion:**

These results show that the combination of BBK and Proton Freezer is suitable for the cryopreservation of iPSC-derived DA neurospheres.

## INTRODUCTION

The transplantation of dopaminergic (DA) neurons is expected as a new treatment for Parkinson’s disease. The proof of concept has been confirmed in previous clinical trials using fetal ventral-mesencephalic (VM) cells [1]. Embryonic stem cells (ESCs) and induced pluripotent stem cells (iPSCs) are expected as alternative donor cells [2–5], and several clinical trials using these cells are ongoing (NCT02452723, NCT03119636, JMA-IIA00384, NCT048-2733).

To make an ESC/iPSC-based therapy a widely available treatment, cryopreservation of the final product is desirable considering the logistics and manufacturing scale. Generally, the *in vivo* survival of cryopreserved cells is less than that of fresh cells. When ESC-derived DA progenitors are frozen as a single cell suspension, the survival ratio in the brain is around 60% that of fresh cells [2]. The transplantation of cell aggregates or neurospheres, instead of a single cell suspension has several advantages, such as more robust viability after the transplantation against anoikis caused by cell dissociation [6, 7], keeping the 3D structure of the donor cells and ease of handling. However, the cryopreservation of cell aggregates is more complicated than that of a single-cell suspension. Namely, it is difficult to achieve an equal distribution of the cryoprotectant (CPA) and temperature throughout the cell aggregates, increasing the risk of intracellular ice formation, which influences cell viability. Indeed, for the transplantation of cryopreserved VM tissues, the survival ratio of DA neurons is reported to be less than 20% that of fresh tissue [8–10]. Therefore, it is critical to find an effective method to cryopreserve the spheres of DA progenitors.

In general, there are two types of cell cryopreservation [11–13]. One is vitrification, an ultra-fast cooling method in which the cells are moved into liquid nitrogen immediately after treatment with a high CPA concentration [14]. It is technically challenging to apply vitrification to the manufacturing of clinical-grade cells, because it requires precise time control [15]. The other type is a slow cooling method, in which cells become frozen at about –1°C/min with a low concentration of CPA, such as 10% dimethyl sulfoxide (DMSO) [16, 17]. In this case, extracellular water freezes first, which increases the osmotic pressure of the extracellular space. The osmotic gap between the intra- and extra-cellular space causes cell dehydration. Consequently, intracellular ice formation can be reduced.

To achieve the cryopreservation of neurospheres towards clinical application, we screened various CPAs and freezing protocols from which we propose the best cryopreservation method for neurospheres to keep high viability and function.

## MATERIALS AND METHODS

### Maintenance and neural differentiation of human iPSCs

This study was approved by the ethical committees of Kyoto University (Kyoto, Japan) and Sumitomo Dainippon Pharma (Osaka, Japan). Human iPSC lines 1231A3 and S17 were used in the study. 1231A3 was generated under feeder-free conditions from the donor’s peripheral blood using episomal vectors. S17 was established by reprogramming the donor’s peripheral blood using Sendai viral vectors (CytoTune-iPS 2.0LG; ID Pharma) under GMP compliance at Sumitomo Dainippon Pharma [18]. Human iPSCs were maintained and differentiated as described in detail [19]. When we began neural differentiation, the iPSCs were dissociated into single cells after 10 min of incubation with 0.5×TrypLE Select and were plated at 5.3×10^5^ cells/cm^2^ onto iMatrix511 (Nippi)-coated plates with differentiation media containing GMEM supplemented with 8% KSR, 0.1 mM MEM nonessential amino acids (all Thermo Fisher Scientific), 1 mM sodium pyruvate (Sigma-Aldrich), and 0.1 mM 2-mercaptoethanol. 0.5×TrypLE Select was prepared by mixing TrypLE select (Thermo Fisher Scientific) and 0.5 mM EDTA/PBS. We added 100 nM LDN193189 (STEMGENT) and 500 nM A83-01 (Wako) to induce neuronal differentiation efficiently. We also added 2*μ*M purmorphamine (Wako) and 100 ng/mL FGF8 (Wako) from day 1 to day 7 and 3*μ*M CHIR99021 (Wako) from day 3 to induce floor plate cells.

### Cell sorting and culture

To apply fluorescence-activated cell sorting, cultured cells were dissociated using 0.5×TrypLE select and stained with PE-conjugated anti-CORIN antibody (100 ng/mL; Catalent/BD) for 20 min. Dead cells and debris were excluded by 7-AAD staining. The analysis was performed using a FACS Aria II or III cell sorter (BD Biosciences) and Gigasort (Cytonome). After cell sorting on culture day 12, the sorted cells were replated on low cell adhesion U bottom 96-well plates (Sumitomo Bakelite) at a density of 3×10^4^ cells per well and in neural differentiation medium containing neurobasal medium supplemented with B27 supplement, 2 mM Glutamax-I (all Thermo Fisher Scientific), 10 ng/mL GDNF, 200*μ*M ascorbic acid, 20 ng/mL BDNF (all Wako), 400*μ*M dbcAMP (Sigma-Aldrich), and 100 nM LDN193189. Unsorted cells were replated at a density of 1.5×10^4^ cells per well in a neural differentiation medium without LDN193189. We changed the medium every 3 days, and 30*μ*M of Y-27632 (Wako) was added to the first medium. For prolonged culture, cells were cultured in the neural differentiation medium.

### Cell transplantation

Animals were cared for and handled according to the Guidelines for Animal Experiments of Sumitomo Dainippon Pharma. Adult male 6-OHDA-lesioned F344 NJcl–rnu/rnu rats (CLEA Japan) were used. Cell transplantation was performed by the stereotactic injection of spheres (A,+1.0; L, –3.0; V, –5.0 and –4.0 from bregma; and TB, 0 (2*μ*L; 200,000 cells/*μ*L); or A,+1.0; L, –3.5 and 2.5; V, –5.5 and –4.5 from bregma; and TB, 0 (4*μ*L; 200,000 cells/*μ*L)) into the right striatum. The number of injected cells was determined by the cell count before cryopreservation. The animals were anesthetized and perfused transcardially with PBS, followed by 4% paraformaldehyde.

### Behavioral analysis

The methamphetamine-induced rotation assay was performed pre-transplantation and every 4 weeks from 8 weeks after the transplantation using video-monitored rotational bowls and video tracking software (EthoVision XT; Noldus). A dose of 2.5 mg/kg of methamphetamine (Sumitomo Dainippon Pharma) was injected intraperitoneally, and the rotations were recorded for 90 min.

### Cryopreservation

Spheres collected on day 28 were placed in cryovials with 1 mL ice-cold cryopreservation medium (see Table 1) and kept on ice until freezing. For the cryopreservation, the vials were transferred into a freezing container (BICELL; NIHON FREEZER), one of three programmed freezers: PDF-150 or 250 (STREX), Cryomed (Thermo Fisher Scientific), or Proton Freezer (Ryoho Freezer Systems). Six cooling profiles (shown in Fig. 3) were used. BICELL was transferred into a deep freezer (–80°C) and kept for more than 4 h. For the programmed freezers, the vials were frozen at –0.5 or –1°C/min until –40°C and then at about – (3–5)°C/min until –80°C. In the shock cooling method, the following steps were taken beginning at –4°C: freezing at –25°C/min until –35°C and then heating at +10°C/min until –12°C. Vials in Proton Freezer were kept in the chamber for 30–60 min until completely frozen. Proton Freezer combines a static magnetic field (SMF), alternating electric field (AEF), and intense airflow. After freezing, the cryovials were stored in the vapor phase of a liquid nitrogen tank. The frozen cells were quickly thawed at 37°C and diluted ten times with neurobasal medium. After supernatant removal, the cells were rinsed with PBS or saline and used for each assay or transplantation. To estimate the cell number after cryopreservation, approximately 50 aggregates were dissociated and counted by a hemocytometer to calculate the cell concentration one day after thawing and before freezing.

**Table 1:** Commercially available xeno-free cryopreservation media tested in this study

Abbreviation	Brand name	Supplier	Major component
SCB	STEM-CELLBANKER	ZENOAQ	10% DMSO
SCB DMSO-free	STEM-CELLBANKER	ZENOAQ	10% Propylene glycol DMSO-free
BBK	Bambanker hRM	NIPPON Genetics	10% DMSO
CS5	Cryostor CS5	BioLife Solutions	5% DMSO
CS10	Cryostor CS10	BioLife Solutions	10% DMSO
SaF	Synth-a-Freeze	Thermo Fisher Scientific	10% DMSO

DMSO, dimethyl sulfoxide.

### Quantitative RT-PCR

Total RNA was extracted using an RNeasy Mini Kit or RNeasy Micro Kit (Qiagen), and cDNA was synthesized using the SuperScript III First-Strand Synthesis System (Thermo Fisher Scientific). Quantitative PCR were carried out with the Fast SYBR Green PCR Master Mix in StepOne (Applied Biosystems). The data were assessed using the delta-Ct method and normalized by the *GAPDH* expression. The primer sequences used are shown in Supplementary Table 1.

### Immunofluorescence studies

For the *in vitro* studies, the cultured cells were fixed with 4% paraformaldehyde. For the *in vivo* studies, fixed frozen brains were sliced at 40*μ*m thickness. The slices were immunologically stained using the free-floating method. The primary antibodies used are listed in Supplementary Table 2. The cells were visualized using a fluorescence microscope (BZX710 and BZX810; Keyence, AxioScan; Zeiss) and one of three confocal laser microscopes (Fluoview FV1200; Olympus, and LSM880 and LSM800; Zeiss). The number of immunoreactive cells was quantified in every 6th section throughout the grafts and corrected using the Abercrombie method. The mean intensity of the IBA1 staining was measured in the graft areas and is shown as a ratio to the contralateral striatum. Image processing and analysis were done using Photoshop (Adobe Systems) and Fiji software.

### Neurite extension assay

Floating spheres on day 28 were plated on 24-well plates coated with iMatrix511 for five days and fixed with 4% paraformaldehyde. The spheres were stained with PE-conjugated anti-PSA-NCAM antibody (1 : 100; Milteny) and visualized using the fluorescence microscope. The area covered by PSA-NCAM positive neurites was measured using Photoshop and WinRoof (Mitani Corporation).

### Electrophysiological analysis

S17-derived DA neurospheres on day 28 were cultured on plates coated with poly-l-ornithine, fibronectin, and laminin (O/F/L) until whole-cell patch-clamp recordings. 1231A3-derived DA neurospheres were dissociated with papain before plating. The cells were maintained in physiological saline solution with the following composition: 125 mM NaCl, 2.5 mM KCl, 2 mM CaCl_2_, 1 mM MgCl_2_, 26 mM NaHCO_3_, 1.25 mM NaH_2_PO_4_, and 17 mM glucose. Patch pipettes were made from borosilicate glass capillaries (GC150TF-10; Clark) and had a 3–4 MW resistance when filled with an internal solution composed of 140 mM KCl, 10 mM HEPES, and 0.2 mM EGTA (pH 7.3). Recordings with a voltage clamp and current clamp were made with a patch-clamp amplifier (Axopatch 200B; Molecular Devices). The giga-seal resistances were in the range of 10–20 G*Ω*. The current signals from the amplifier were filtered at 1 kHz and stored and analyzed on a 64-bit computer (Cooler Master). All experiments were performed at room temperature.

### Multielectrode array (MEA)

Floating spheres on day 28 were cultured on laminin-coated HD-MEA chips (MaxWell Biosystems) until the recordings. The recordings were performed using the Activity Scan Assay and Network Assay modules on MaxLab Live software (MaxWell Biosystems). The entire HD-MEA was scanned using the Activity Scan Assay module, and electrodes on the spheres were selected. Spontaneous neuronal activities were recorded from the selected electrodes for 30 sec using the Network Assay module. After inactive electrodes (< 0.5 Hz) were removed, the mean values of spike amplitudes were calculated for each sphere.

### Dopamine release assay

Floating spheres on day 28 were cultured on O/F/L-coated 12- or 24-well plates for 28 days, washed twice with low KCl (4.7 mM) solution, and incubated in low KCl solution for 15 min. The medium was subsequently replaced with high KCl solution (60 mM) for 15 min. The solution was then collected, and the concentration of dopamine was determined by LC-MS/MS using QTRAP6500 or Triple Quad 6500 (AB Sciex) coupled to Nexera X2 (Shimadzu). Cells that remained on the plate were harvested in PBS and sonicated. The DNA concentration of the cell lysate was measured using the Quant-iT dsDNA Assay Kit (Thermo Fisher Scientific) and used to compensate the dopamine concentration.

### Statistical analysis

Statistical analyses were performed using a commercially available software package (GraphPad Prism 8; GraphPad Software). Data were analyzed by a one-way ANOVA and Tukey’s *post hoc* analysis groups, an unpaired *t*-test, or a two-way ANOVA with Tukey’s multiple comparisons test, as indicated in the figure legends.

## RESULTS

### Induction of midbrain DA progenitors from iPSCs

**Fig. 1:**
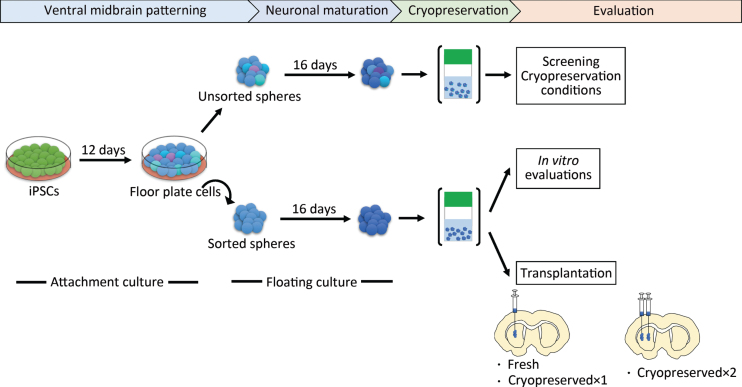
Schematic overview of the protocol steps. Cryopreserved×1 and Cryopreserved×2 are defined in the main text. iPSCs, induced pluripotent stem cells.

We induced DA progenitors from a research-grade human iPSC line (1231A3) based on a protocol with dual SMAD inhibition and floor plate induction [20]. To screen for the best cryopreservation conditions, we did not employ cell sorting to simplify the procedure (Fig. 1). The induced spheres were mainly comprised of DA progenitors (FOXA2/LMX1A; 86.1±5.1%) and contained some neural stem cells (SOX1; 4.7±1.6% and PAX6; 2.7±0.8%) on day 28 (Supplementary Figure 1).

### Cryopreservation with Bambanker hRM (BBK) yields good post-thaw recovery and neurite extension

**Fig. 2:**
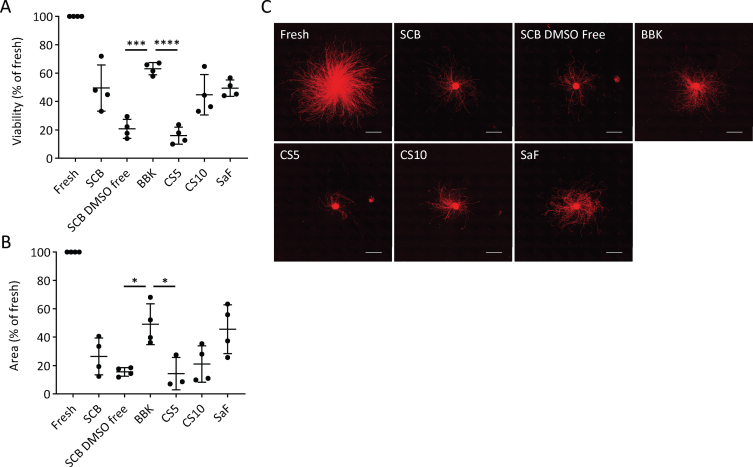
Effects of cryopreservation media on iPSC-derived neurospheres (A): Viability and (B): neurite extension of spheres from unsorted cells cryopreserved at –0.5°C/min on day 28 using the cryopreservation media shown in table 1 (*n* = 4). Viability and neurite extensions were analyzed on day 1 (24 hours) and day 5, respectively. One-way ANOVA with Tukey’s multiple comparisons test; ^*^p < 0.05, ^***^p < 0.001, ^****^p < 0.0001 versus Bambanker hRM. C: Immunostaining of neurites for PSA-NCAM. Scale bars, 1 mm. Data are shown as means±SD.

First, we determined the best CPA among six clinically available ones (Table 1). After freezing the day-28 spheres at –0.5°C/min in each CPA, we thawed them and compared the cell viability and neurite extensions. We chose day 28, because we are transplanting fresh day-28 to -30 cells for Parkinson’s disease patients in our clinical trial [19]. Some cells died during the freeze/thaw procedure due to apoptosis and necrosis [21], but the survived cells started to proliferate within 24 to 48 h after thawing [22–24]. Previous report indicated that measuring cell viability at 24 h post-thawing is essential to evaluate the quality and efficiency of a cryopreservation process [25]. Therefore, we examined the percentage of viable cells (viability) at 24 h, finding that BBK (63±4%) gave a significantly higher percentage than STEM-CELL BANKER DMSO free (SCB DMSO-free; 21±7%) or CryoStor CS5 (CS5; 16±6%) (Fig. 2A). SCB (49±16%), SaF (49±6%), and CS10 (45±14%) ranked second best in terms of cell recovery without any significant difference among them.

Neurite extensions are diminished by cell damage, such as neurotoxic injury and disease-related genetic aberration [26–28]. Therefore, we measured the area covered by neurites extended from the spheres five days after plating on day 28 to evaluate cell function. With BBK, the area was 49±14% that of fresh spheres, a percentage significantly larger than with SCB DMSO-free or CS5 (Fig. 2B, C). Adding that BBK has already been registered in a Japanese drug master file, we concluded that BBK is the best CPA to cryopreserve DA progenitor spheres.

### Proton Freezer provides the optimal freezing condition

**Fig. 3:**
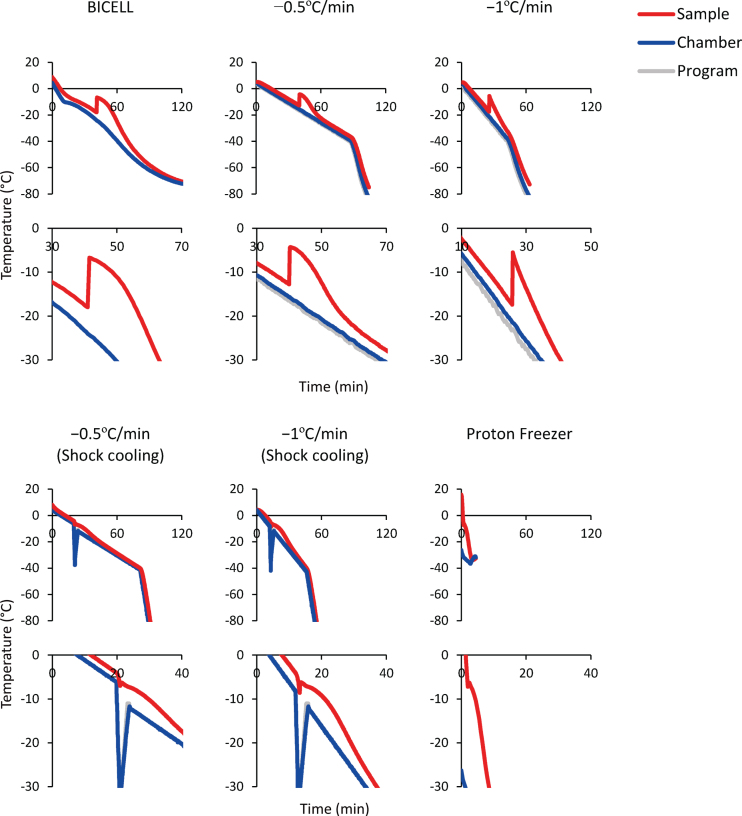
Time-temperature curves of the sample (red line), freezing chamber (blue line), and program (gray line). Bambanker hRM was used as the sample. The temperature change caused by latent heat release is magnified in the lower graphs.

Next, to determine the optimal freezing protocol, we froze the spheres in BBK by six different cooling conditions and two equilibration times (Fig. 3). Intracellular ice formation is a significant cause of cell death, and the control of cell dehydration and ice nucleation is a critical parameter that affects cell viability. The slower the cells are cooled, the stronger the cell dehydration and less intracellular ice. On the other hand, excessive cellular dehydration increases the solute concentration and damages cells [29]. Therefore, an optimal cooling rate is needed. Furthermore, during the freezing process, the CPA temperature drops below the freezing point before ice is formed (i.e., supercooling). When freezing starts, the temperature rises due to the release of latent heat and returns to the freezing point. In this process, intracellular freezing occurs because the cells freeze at once without dehydration [30, 31]. Therefore, it is essential to minimize supercooling.

BICELL is a conventional freezing container. We tested BICELL and two cooling speeds, –0.5°C/min and –1°C/min, using a programmed freezer. Next, we tried the shock cooling method. This method induces ice nucleation by a transient temperature drop and suppresses supercooling [32]. The final candidate was Proton Freezer. This freezer can rapidly cool the sample from –4°C to –30°C at –5°C/min, an intermediate speed between conventional slow cooling and vitrification. In addition, Proton Freezer includes a SMF and AEF, which align the orientation of water molecules, minimizing the ice crystals and preventing cell destruction. Finally, because the equilibration time in CPA before freezing is another critical issue to be determined, we compared 15- and 60-minute equilibration times.

**Fig. 4:**
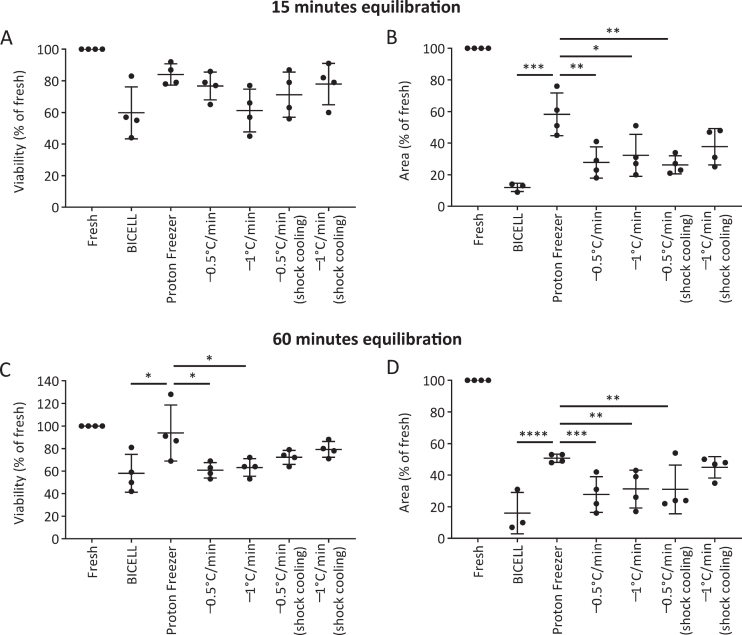
Effects of the freezing program and equilibration time on iPSC-derived neurospheres. A, C) Viability and (B, D) neurite extensions of spheres from unsorted cells cryopreserved on day 28 under the different freezing programs after 15 min (A, B) and 60 min (C, D) equilibration in Bambanker hRM (*n* = 4). Viability and neurite extensions were analyzed on day 1 (24 h) and day 5, respectively. One-way ANOVA with Tukey’s multiple comparisons test; ^*^p < 0.05, ^**^p < 0.01, ^***^p < 0.001, ^****^p < 0.0001 versus Proton Freezer. Data are shown as means±SD.

We evaluated the viability and neurite extension of cells cryopreserved in BBK. At 15 minutes equilibration, there was no significant difference in viability at 24 hours between the six conditions (Fig. 4A). On the other hand, Proton Freezer showed more robust neurite extensions than the other conditions, suggesting only minor damage to the cells (Fig. 4B). At 60 min equilibration, Proton Freezer resulted in higher cell viability and neurite extensions than all other conditions (Fig. 4C, D). Neither cell viability nor neurite extensions showed a significant difference between the 15- and 60-minute equilibrations in BBK (Supplementary Figure 2).

Based on these results, we concluded that the combination of BBK and Proton Freezer is suitable for the cryopreservation of neurospheres.

### Cryopreserved iPSC-derived neurospheres maintain their characteristics and functions

**Fig. 5:**
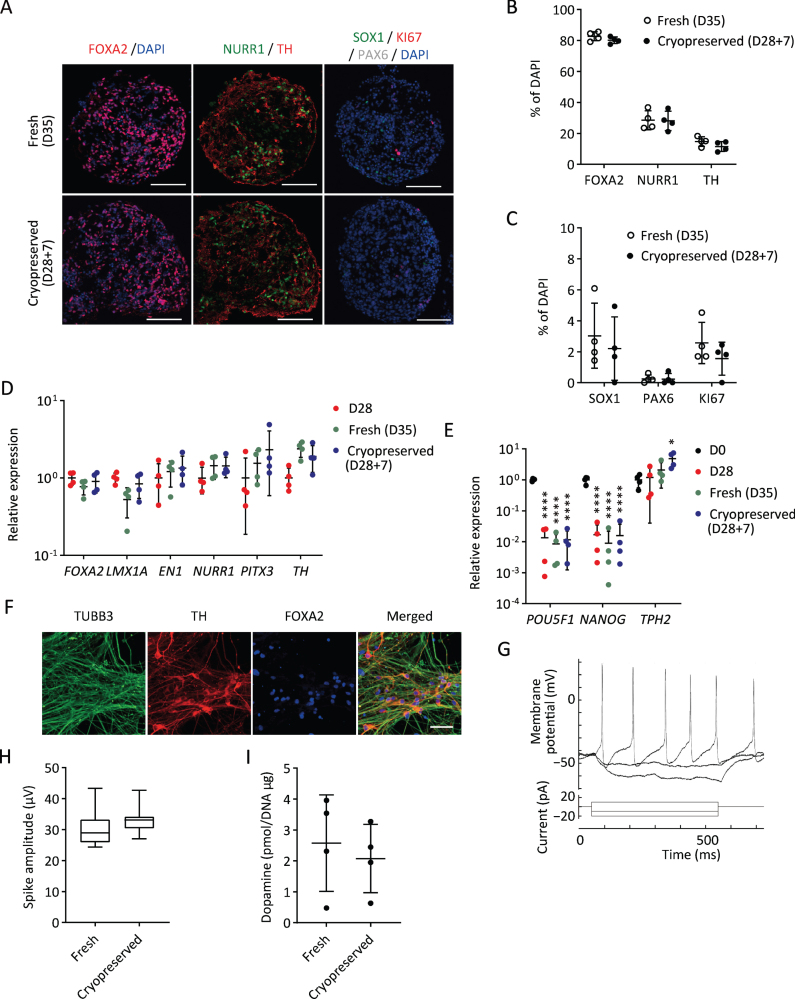
Characterization of cryopreserved spheres derived from S17 *in vitro*. A) Immunostaining of the spheres on day 35. FOXA2/DAPI (left), NURR1/TH (center), and SOX1/KI67/PAX6/DAPI (right). Scale bars 100*μ*m. B, C) The percentages of FOXA2^+^, NURR1^+^, TH^+^ (B) and SOX1^+^, PAX6^+^ and KI67^+^ (C) cells per total cells on day 35 (*n* = 4). D, E) Gene expressions of the spheres relative to GAPDH measured by quantitative RT-PCR (*n* = 4). D28, cells cultured for 28 days; D28 + 7, cells cultured for 7 days after 28 days cryofreezing; D35, fresh cells cultured for 35 days. The expression level of D28 (D) and undifferentiated cells (D0) (E) was set to 1. There were no significant differences between D35 and D28 + 7 by one-way ANOVA with Tukey’s multiple comparisons test (D). One-way ANOVA with Tukey’s multiple comparisons test; ^*^p < 0.05, ^****^p < 0.0001 versus D0 (E). F) Immunostaining of post-thawed iPSC-derived DA neurons for TUBB3, TH, and FOXA2 on day 49. Scale bars, 50*μ*m. G) Representative induced action potentials of post-thawed iPSC-derived DA neurons on day 68. H) Box plots display the value in each sphere of spike amplitudes on day 42. I) The dopamine release on day 56 induced by high potassium stimulation (*n* = 4). Data are shown as means±SD.

Next, we investigated the characteristics and functions of cryopreserved spheres derived from a clinical-grade human iPSC line (S17). We sorted the cells expressing CORIN (a floor plate marker in the developing brain) on day 12 to enrich DA progenitors, cultured the sorted cells as floating spheres until day 28, and froze them in BBK using Proton Freezer (Fig. 1). We thawed the spheres and found that the cell viability and the area covered by the neurites were 63±19% and 35±21%, respectively, compared to those of fresh spheres. To determine DA neurons, the expressions of related proteins and genes were examined seven days after thawing. Immunocytochemistry revealed that the cryopreserved spheres (D28 + 7) showed similar expressions of a floor plate marker, FOXA2, and DA neuron markers, NURR1 and TH (Fig. 5A, B), compared to fresh spheres (D35). There was no difference in the expression of two neural stem cell markers, SOX1 and PAX6, and a proliferating cell marker, KI67 (Fig. 5A, C). Quantitative PCR analyses revealed that the expression levels of several DA markers, including *FOXA2*, *LMX1A*, *EN1*, *NURR1*, *PITX3*, and *TH*, were unaffected by the cryopreservation method (Fig. 5D). In all conditions, the expression levels of the pluripotent markers *POU5F1* and *NANOG* were significantly reduced compared to undifferentiated iPSCs (Fig. 5E).

To confirm the DA function of the spheres, we conducted electrophysiological analysis and dopamine release measurements. When the cryopreserved spheres were cultured on the plate for further maturation, most of the cells differentiated into TH^+^TUBB3^+^ double positive DA neurons on day 49 (Fig. 5F). Repeated action potentials were detected by whole-cell current-clamp recording on day 68 (Fig. 5G). We also measured spontaneous electrophysiological activity by MEA. No difference was observed in spike amplitudes between fresh and cryopreserved cells (Fig. 5H). Dopamine secretion was measured by LC/MS/MS. The amount of dopamine released by the cryopreserved spheres on day 56 was comparable to that of the fresh spheres (Fig. 5I).

These results suggest that cryopreserved spheres can differentiate into mature and functional DA neurons as fresh spheres do. Regarding the expression of markers and functional activities, similar results were obtained using another iPSC line (1231A3) (Supplementary Figure 3).

### Cryopreserved spheres improve the behavior of 6-OHDA lesioned rats

**Fig. 6:**
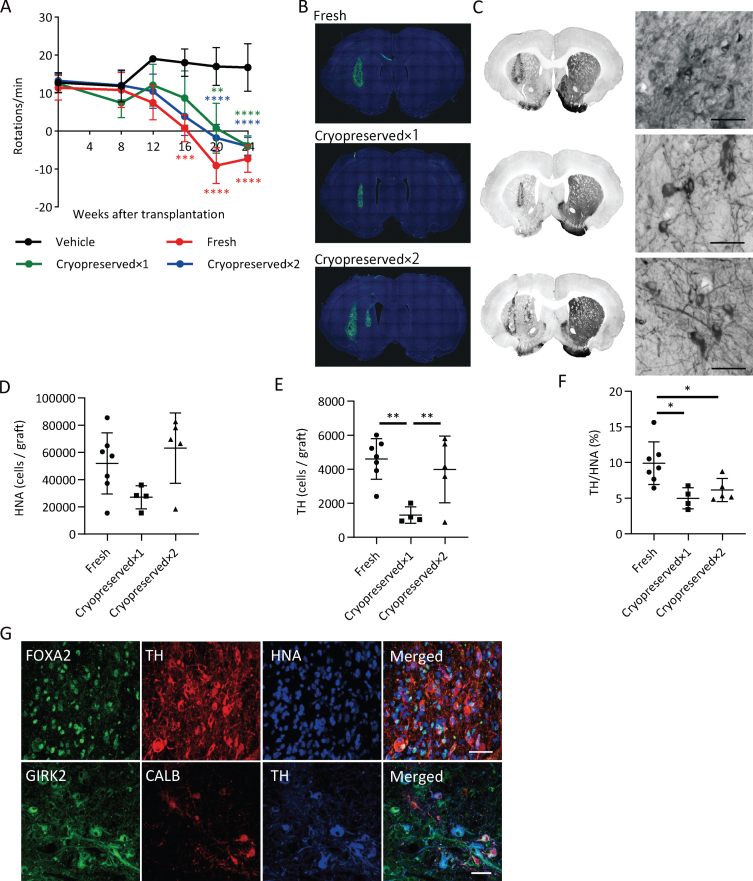
Graft survival and function of cryopreserved spheres. A) The methamphetamine-induced rotations of rats that received the grafts. (*n* = 4–7). Two-way ANOVA with Tukey’s multiple comparisons test; ^**^p < 0.01, ^***^p < 0.001, ^****^p < 0.0001 versus vehicle group. B) Immunostaining of representative grafts for HNA (green) and DAPI (blue). C) DAB staining of representative grafts for TH. The right panels are magnified images of the left panels. Scale bars, 50*μ*m. D) The number of survived HNA^+^ cells in the grafts (*n* = 4–7). E) Number of survived TH^+^ cells in the grafts (*n* = 4–7). F) The percentages of TH^+^ cells per survived human cells (*n* = 4–7). One-way ANOVA with Tukey’s multiple comparisons test; ^*^p < 0.05, ^**^p < 0.01 (D–F). G) Immunostaining of the grafts derived from cryopreserved cells for FOXA2, TH, and HNA (upper) and GIRK2, CALB, and TH (lower). Scale bars, 50*μ*m. Data are shown as mean±SD.

We transplanted fresh or cryopreserved spheres into 6-OHDA-lesioned rats and investigated cell survival and pharmacological efficacy. We set two dose groups for the cryopreserved spheres: cryopreserved×1 (4×10^5^ cells) and cryopreserved×2 (8×10^5^ cells). We analyzed the methamphetamine-induced rotation every four weeks. Each group with the cell transplantation showed improved abnormal behavior after 20 weeks (Fig. 6A).

An immunofluorescence study at 24 weeks revealed that the numbers of survived human cells (HNA^+^ cells) in the graft were 51,944±22,462 (13.0±5.6% of injected cells), 27,046±8,490 (6.8±2.1% of injected cells) and 63,108±25,834 (7.9±3.2% of injected cells) for the fresh, cryopreserved×1 and cryopreserved×2 groups, respectively (Fig. 6B, D), and the numbers of TH^+^ cells in the graft were 4,601±1,189 (1.2±0.3% of injected cells), 1,306±480 (0.3±0.1% of injected cells) and 3,988±1,961 (0.5±0.2% of injected cells), respectively (Fig. 6C, E). These results suggest that the survival rate of TH^+^ neurons from cryopreserved spheres is about half that compared to fresh ones. The percentages of TH^+^ cells per survived human cells in the graft were relatively low in cryopreserved spheres compared to fresh ones (Fig. 6F). TH^+^ cells exhibited neurite outgrowth in every group (Fig. 6C) and were positive for FOXA2 (Fig. 6G). Finally, we observed GIRK2^+^ A9-subtype DA neurons had a large cell body with good arborization and Calbindin (CALB)^+^ A10-subtype DA neurons had a relatively small cell body in the grafts (Fig. 6G).

To examine the immune response to the grafts, we performed staining for IBA1, a marker of microglia (Supplementary Figure 4). Activated microglia were observed around the grafts, but there was no difference in the density of microglia between the grafts of fresh and cryopreserved cells.

## DISCUSSION

Here we demonstrated the advantage of combining cryopreservation medium BBK and Proton Freezer to cryopreserve iPSC-derived neurospheres for cell transplantation therapies. Several groups have reported the effects of cryopreservation on neurospheres (Supplementary Table 3). They all found good cell viability *in vitro*, but the *in vivo* survival and function in the brain are unknown [33–36]. In most cases, the spheres were cryopreserved at – (0.5–1)°C/min using DMSO as a CPA based on previous reports for the cryopreservation of neural tissues [37]. Cryopreserved VM tissues, however, often showed poor survival of DA neurons after transplantation [8–10]. Here, we established a novel, clinically applicable cryopreservation method suitable for iPSC-derived neurospheres. As shown in the previous reports about the cryopreservation of dissociated cells [2, 25, 38], the cryopreserved spheres were also equivalent to fresh spheres in the expression of DA-specific markers, dopamine secretion, and electrophysiological activity. Following their grafting into the brains of 6-OHDA lesioned rats, abnormal rotational behavior was improved.

Recently, various xeno-free, serum-free, and cGMP grade CPAs have been launched for clinical use. In the present study, iPSC-derived neurospheres cryopreserved using BBK containing 10% DMSO showed higher cell viability than other CPAs with 5% DMSO or DMSO-free (Fig. 2). For the cryopreservation of neural stem cell spheres, a higher DMSO concentration than for single-cell suspensions is suitable [39]. The reason is the difficulty in achieving uniform CPA diffusion and cell dehydration in the spheres. For most cell and tissue types, around 10% DMSO shows good cell viability [40–42], which is consistent with our results.

Next, we focused on the cooling program and revealed that Proton Freezer enabled high viability and favorable neurite extensions (Fig. 4). Proton Freezer freezes samples by a combination of weak SMF (1–200 mT), AEF in the region of radio waves (0.2–1 MHz), and cold air [43]. The application of a magnetic field (MF) and electric field (EF) to the cryopreservation has been studied and developed mainly for food preservation and used to freeze various foods, including fish and meat, and more recently biomaterials. The effects of a MF and EF on cryopreservation vary depending on the intensities, frequencies, solutions, etc., and the precise mechanisms underlying them are not entirely elucidated [44]. Previous papers suggest that SMF increases the hydrogen bonds between water molecules and makes more ordered and stable distributed water clusters, leading to the promotion of ice nucleation and suppression of supercooling [45–47]. AEF affects the vibration and orientation of water molecules and changes ice formation properties [46, 48]. The influence of AEF on ice formation is highly dependent on its frequency. When freezing in 0.9% NaCl solution, a 500 kHz frequency minimized supercooling [49], but AEF at 20 kHz or radio frequencies induced tiny ice crystals [50, 51]. Thus, Proton Freezer can minimize ice crystals by SMF and AEF. SMF aligns water molecules and prevents ice crystals from growing larger, while AEF vibrates water molecules and accelerates ice nucleation. These effects of SMF and AEF possibly improved the cell viability of iPSC-derived neurospheres.

6-OHDA lesioned rats showed complete recovery of methamphetamine-induced abnormal rotations five months after the transplantation of either cryopreserved or fresh cells (Fig. 6A). This observation suggests that cryopreserved cells in the spheres retained DA neuron function even in the brain. When comparing TH^+^ cell survival per injected cell, the cryopreserved spheres showed about 50% survival compared to fresh ones (Fig. 6E). This rate is better than previous reports, which showed less than 20% TH^+^ cell survival [8–10]. Notably, the percentage of TH^+^ cells per survived human cells in the graft was significantly reduced in cryopreserved spheres compared to fresh ones (Fig. 6F). Cryopreservation transiently decreases cell metabolism and biochemical reactions [24], and cryopreserved cells take a longer time for maturation after transplantation than fresh cells due to the freezing damage [52]. Considering there was no significant difference in the number of survived cells between the grafts of fresh and cryopreserved spheres (Fig. 6D), we concluded that maturation of the cryopreserved spheres was delayed.

In addition, the lower survival rate leads to the concern that cryopreserved cells may induce a more robust immune response than fresh cells because more dead cells may become present the brain. However, there was no significant difference in the immune response by the host brain (Supplementary Figure 4). This observation suggests that the possible increase in dead cells due to cryopreservation causes no significant impact on the immune response because in both cases, cryopreserved cells and fresh cells, most of the cells die after transplantation.

In conclusion, this study developed a cryopreservation method suitable for iPSC-derived DA neurospheres by introducing a unique cooling method. The cryopreserved cells maintain function as DA neurons, including pharmacological activity. Along with the freezing processes, further improvements may be possible by optimizing other parameters such as the thaw rate, cell density in the vials, freezing volume, and cryovial type (material, thickness, etc.). Importantly, our approach enables off-the-shelf availability of large-scale iPSC-derived neurospheres and can be applied to the cryopreservation of other types of tissues or cell aggregates.

## Supplementary Material

Supplementary Material
