# Supplementary material for: Cryopreservation of Induced Pluripotent Stem Cell-Derived Dopaminergic Neurospheres for Clinical Application
Source: J Parkinsons Dis. 2022 Apr 5;12(3):871–84. doi: 10.3233/JPD-212934 (PMC9108593; doi:10.3233/JPD-212934)

Supplementary Material

Cryopreservation of Induced Pluripotent Stem Cell-Derived Dopaminergic Neurospheres for Clinical Application

Supplementary Table 1. List of primers for quantitative RT-PCR.

**Gene**	**Forward**	**Reverse**
POU5F1	AGACCATCTGCCGCTTTGAG	GCAAGGGCCGCAGCTT
NANOG	GGCTCTGTTTTGCTATATCCCCTAA	CATTACGATGCAGCAAATACGAGA
FOXA2	TTCAGGCCCGGCTAACTCT	AGTCTCGACCCCCACTTGCT
LMX1A	GATCCCTTCCGACAGGGTCTC	GGTTTCCCACTCTGGACTGC
EN1	TGGGTGTACTGCACACGTTATTC	GGAACTCCGCCTTGAGTCTCT
NURR1	CGAAACCGAAGAGCCCACAGGA	GGTCATAGCCGGGTTGGAGTCG
PITX3	GGGCCAGGAGCACAGCGACTCA	GCTGCCGCCGCTGCTTCTTTTT
TH	GCAGTTCTCGCAGGACATTG	CGGCACCATAGGCCTTCA
TPH2	TCAGCTACTTGGCAGCTCAAC	CTTGCCACTTTCGGTAGCAG
GAPDH	GGTCGGAGTCAACGGATTTG	TCAGCCTTGACGGTGCCATG

Supplementary Table 2. List of primary antibodies.

**Antibody**	**Species**	**Dilution**	**Supplier**	**Catalog number**
TH	Rabbit	1:400	Millipore	AB152
NURR1	Mouse	1:300	Perseus Proteomics	PP-N1404-00
NURR1	Mouse	1:1000	Donated by the KAN laboratory	
FOXA2	Goat	1:500	R&D systems	AH2400
SOX1	Goat	1:100	R&D systems	AF3369
PAX6	Mouse	1:500	BD Pharmingen	561462
KI67	Rabbit	1:1000	Novocastra	NCLKi67P
KI67	Rabbit	1:1000	Abcam	ab16667
TUBB3	Mouse	1:400	Covance	MMS-435P
HNA	Mouse	1:500	Millipore	MAB1281
IBA1	Rabbit	1:500	WAKO	019-19741

Supplementary Table 3. Summary of techniques for cryopreservation of neurospheres.

Cell source	Cryoprotectant	Freezing program	Results	Reference
Mouse ESC-derived NPC	10% DMSO + 90% FBS or 10% DMSO + 90% HypoThermosol FRS	Cool at −(0.4–0.6)°C/min (Freezing container)	>85% viability and 45% recovery of viable cells. Fresh and cryopreserved cells yielded similar neural marker expressions. Cryopreservation slightly increased the differentiation into neurons and astrocytes.	[32]
Human iPSC-derived DA neuron	10% DMSO + 30% KSR in medium	Slow freezing	71% viability. Correctly patterned midbrain DA neurons co-expressing LMX1A, FOXA2, and TH.	[33]
Human iPSC-derived NSC/NPC	STEM-CELLBANKER	CAS freezer<br>Hold at −7°C for 15 min<br>Plunge to −70°C	>60% viability in the best condition. No significant differences in proliferation ability and neural marker expressions between fresh and cryopreserved cells. Maturation delay was observed in cryopreserved cells.	[34]
Mouse MGE (E12.5)	10% DMSO in medium	Cool at −1°C/min (Freezing container)	92.2% viability and 29.6% recovery of viable cells. Neurosphere formation ability was not maintained.	[35]

ESC, embryonic stem cell; NPC, neural progenitor cell; DMSO, dimethyl sulfoxide; FBS, fetal bovine serum; DA, dopaminergic; iPSC, induced pluripotent stem cell; NSC, neural stem cell; MGE, medial ganglionic eminence

Supplementary Figure 1. Characterization of CORIN-unsorted spheres. A) Immunostaining of the spheres on day 28. LMX1A, FOXA2, and DAPI (upper), NURR1, TH, and DAPI (middle), and SOX1, KI67, PAX6, and DAPI (lower). Scale bars 100 μm. B, C) The percentages of FOXA2^{+}/LMX1A^{+}, NURR1^{+}, and TH^{+} (B) and SOX1^{+}, PAX6^{+}, and KI67^{+} (C) cells per total cells (n = 4).

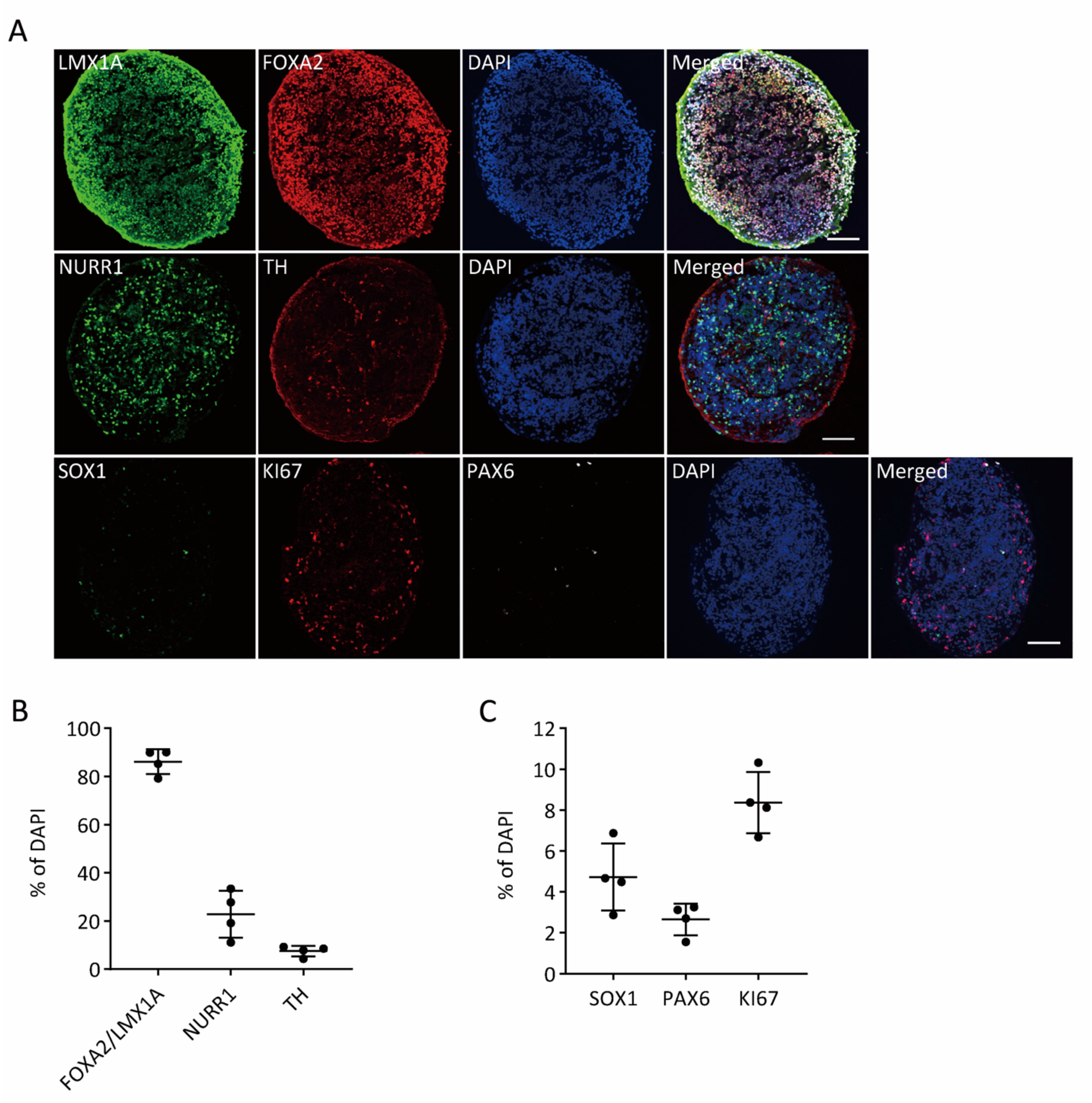

Supplementary Figure 2. Effects of equilibration time on iPSC-derived neurospheres. A) Viability and (B) neurite extension of spheres from unsorted cells cryopreserved in Proton Freezer after 15 and 60 min of equilibration in Bambanker hRM (n = 4). The data are taken from Fig. 4, analyzed by unpaired t-test (ns, not significant), and shown as means ± SD.

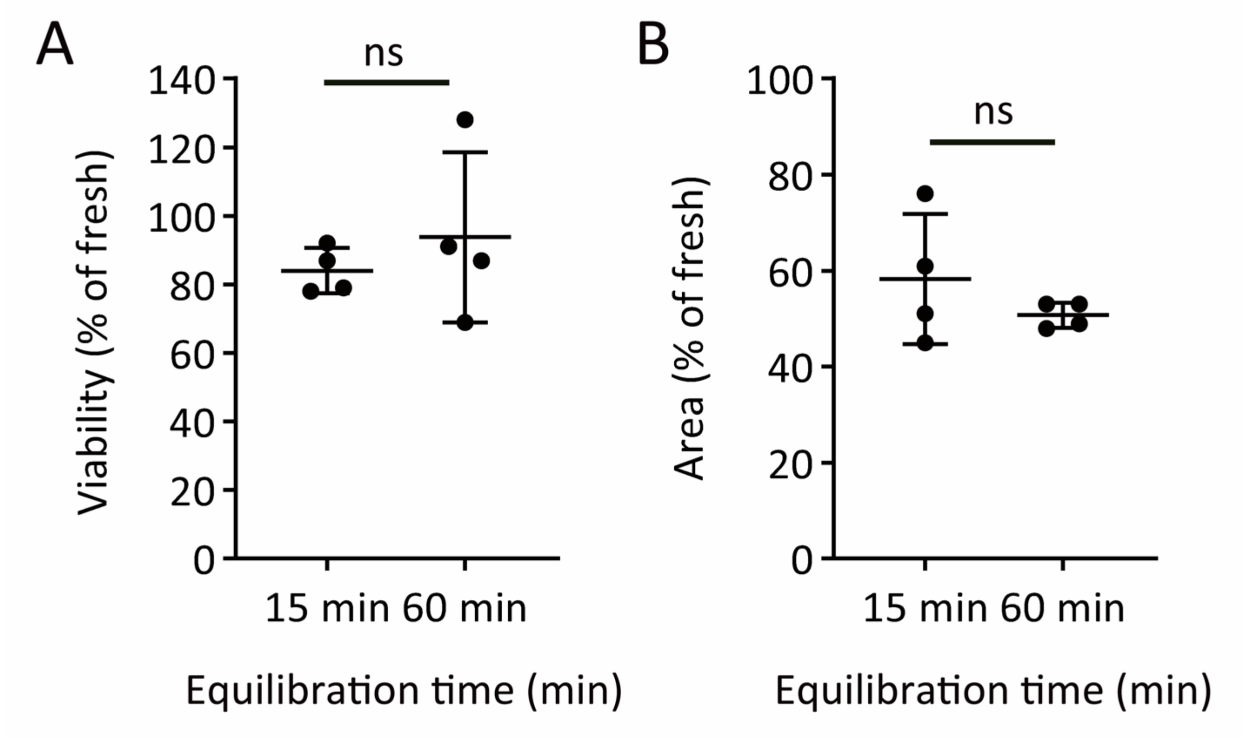

Supplementary Figure 3. Characterization of cryopreserved spheres derived from 1231A3 *in vitro*. A) Immunostaining of the spheres on day 35. FOXA2/DAPI (left), NURR1/TH (center), and SOX1/KI67/PAX6/DAPI (right). Scale bars, 100 μm. B, C) The percentages of FOXA2$^+$, NURR1$^+$, and TH$^+$ (B) and SOX1$^+$, PAX6$^+$, and KI67$^+$ (C) cells per total cells on day 35 (n = 4). D, E) The gene expression of the spheres relative to GAPDH measured by quantitative RT-PCR (n = 4). D28, cells cultured for 28 days; D28+7, cells cultured for 7 days after 28 days cryofreezing; D35, fresh cells cultured for 35 days. The expression levels of day 28 (D) and undifferentiated cells (D0) (E) was set to 1. There were no significant differences between D35 and D28+7 by one-way ANOVA with Tukey's multiple comparisons test (D). One-way ANOVA with Tukey's multiple comparisons test; ****$p < 0.0001$ versus D0 (E). F) Immunostaining of post-thawed iPSC-derived DA neurons for TUBB3, TH, and DAPI on day 50. Scale bars, 50 μm. G) Representative induced action potentials of post-thawed iPSC-derived DA neurons on day 49. H) The results of dopamine release induced by high potassium stimulation on day 56 (n = 3). Data are shown as means ± SD.

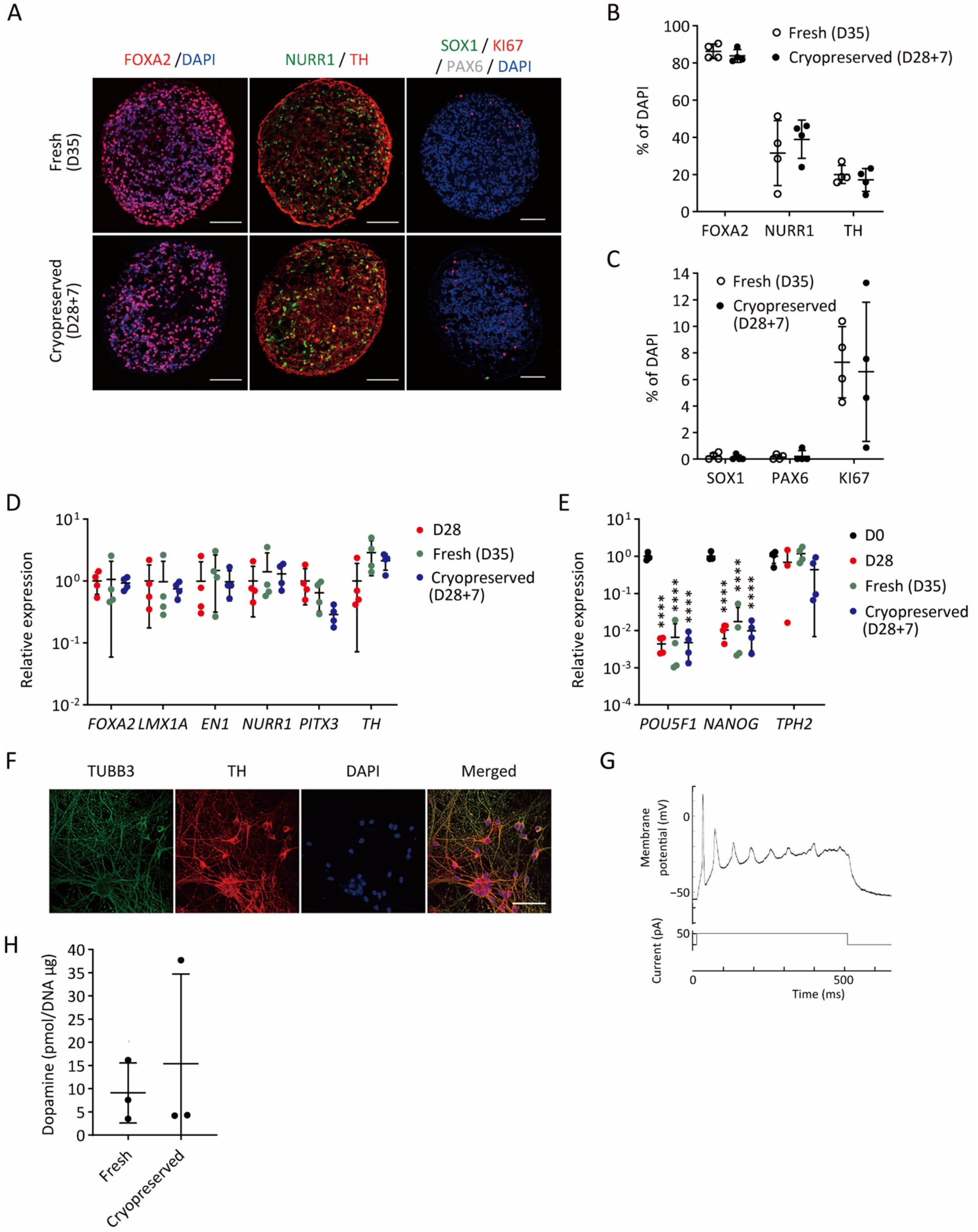

A
FOXA2 / DAPI
NURR1 / TH
SOX1 / KI67 / PAX6 / DAPI
Fresh (D35)
Cryopreserved (D28+7)
B
% of DAPI
Fresh (D35)
Cryopreserved (D28+7)
FOXA2
NURR1
TH
C
% of DAPI
Fresh (D35)
Cryopreserved (D28+7)
SOX1
PAX6
KI67
D
Relative expression
D28
Fresh (D35)
Cryopreserved (D28+7)
FOXA2
LMX1A
EN1
NURR1
PITX3
TH
E
Relative expression
D0
D28
Fresh (D35)
Cryopreserved (D28+7)
POU5F1
NANOG
TPH2
F
TUBB3
TH
DAPI
Merged
G
Membrane potential (mV)
Current (pA)
Time (ms)
H
Dopamine (pmol/DNA μg)
Fresh
Cryopreserved

Supplementary Figure 4. A) Immunostaining of representative grafts for IBA1. The right panels are magnified images of the frames in the left panels. Scale bars, 50 µm. B) Adjusted mean intensity of IBA1 in the graft areas. The values are expressed as a ratio to the contralateral striatum. There were no significant differences between fresh, cryopreserved×1, and cryopreserved×2 by one-way ANOVA with Tukey's multiple comparisons test.

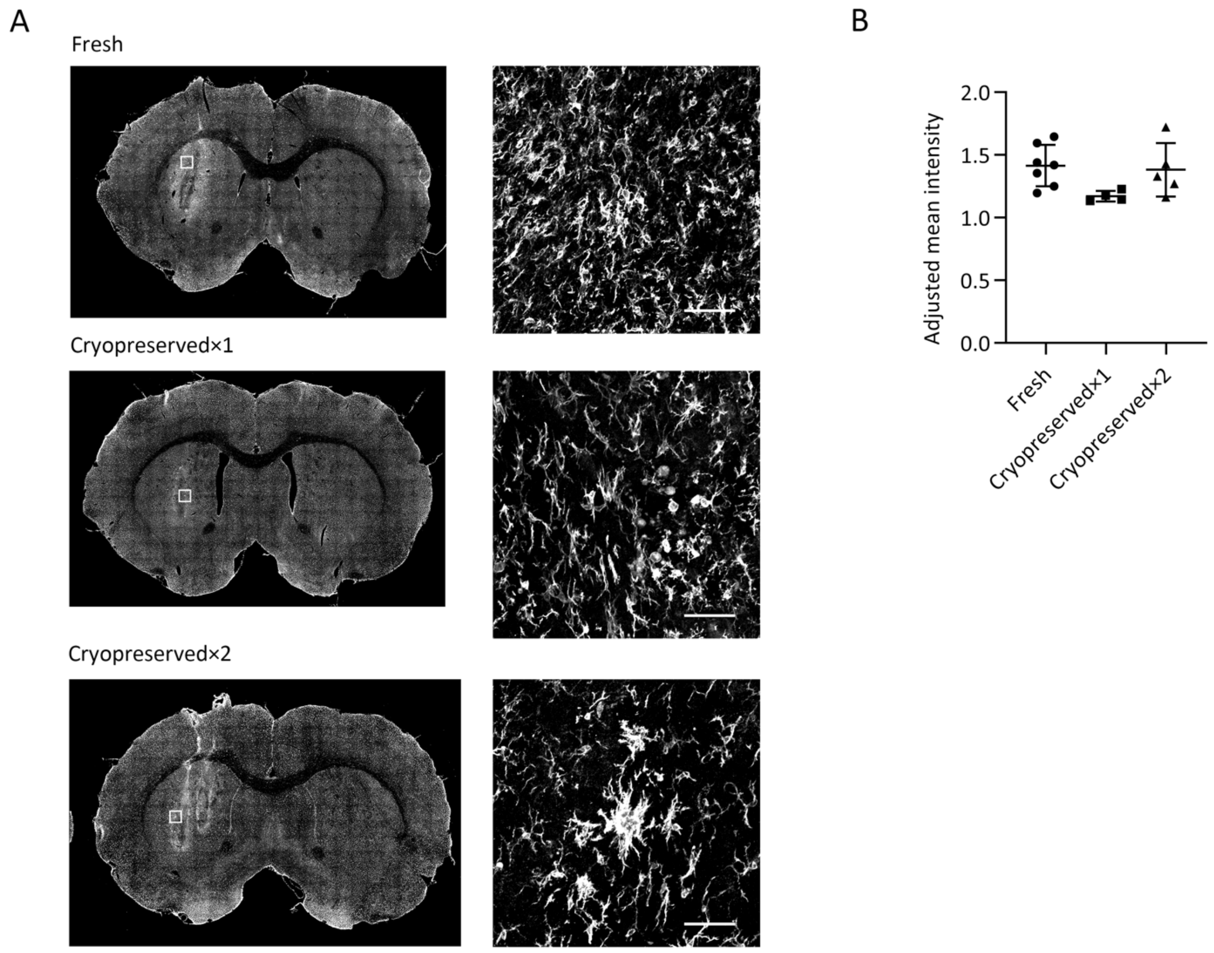

Supplement: Supplementary Material [file jpd-12-jpd212934-s001.pdf]
